# Characterization of four vaccine-related polioviruses including two intertypic type 3/type 2 recombinants associated with aseptic encephalitis

**DOI:** 10.1186/s12985-016-0615-2

**Published:** 2016-09-27

**Authors:** Jiansheng Liu, Haihao Zhang, Yilin Zhao, Longhui Xia, Chen Guo, Huai Yang, Na Luo, Zhanlong He, Shaohui Ma

**Affiliations:** 1 Institute of Medical Biology, Chinese Academy of Medical Sciences, and Peking Union Medical College (CAMS & PUMC), 935 Jiao Ling Road, Kunming, Yunnan Province 650118 People’s Republic of China; 2 Yunnan Key Laboratory of Vaccine Research Development on Severe Infectious Disease, Kunming, 650118 People’s Republic of China

**Keywords:** Vaccine-related polioviruses (VRPVs), Aseptic encephalitis, Intertypic recombinant

## Abstract

**Background:**

Four vaccine-related polioviruses (VRPV) were isolated from aseptic encephalitis cases in Yunnan, China in 2010. The genomic sequences of these VRPVs were investigated to gain a better understanding of their molecular characteristics.

**Methods:**

Molecular typing was performed by amplification and sequencing of the VP1 region. The genomic sequences of the four VRPV3 strains were compared to vaccine strain and wild strain sequences to study genetic drift and recombination.

**Results:**

All four isolates could be entirely neutralized by polyclonal poliovirus 3 (PV3) antisera but not by PV1 and PV2 antisera and displayed a temperature-sensitive phenotype. The genomic sequences of all four isolates contained two Sabin 3-specific attenuating mutations at nucleotides 472(C → T) and 2034(C → T), but a third Sabin 3-specific attenuating mutation at position 2493 (T → C) had reverted back to a T. Recombination analyses showed RF108/YN/CHN/2010 and RF134/YN/CHN/2010 strain recombined with Sabin 2 at the 3′-end of the 2C to 3′-untranslated region (3′-UTR) and at the 5′-end of the 3D to 3′-UTR, respectively.

**Conclusion:**

Four VRPV3 strains including two type 3/type 2 intertypic recombinants were identified. The recombination of Sabin vaccine strains with other Sabin serotypes or human enterovirus C species could be a critical factor in the potential of emerging viruses and related disease outbreaks. Therefore, it is essential to be persistent in the surveillance of EVs (including PV).

**Electronic supplementary material:**

The online version of this article (doi:10.1186/s12985-016-0615-2) contains supplementary material, which is available to authorized users.

## Background

Polioviruses (PVs) are members of the human enterovirus C species (HEV-C) of the *Enterovirus* genus in the *Picornaviridae* family, which are small, non-enveloped, positive single-stranded RNA viruses. The genome of PV is approximately 7500 nucleotides (nt) in length and contains an open reading frame (ORF) flanked by a 5′-untranslated region (UTR) and a 3′-UTR. The ORF is translated into a polyprotein and is then cleaved into four capsid proteins (VP1 to VP4) and seven nonstructural proteins (2A, 2B, 2C, 3A, 3B, 3C, and 3D). The oral poliovirus vaccine (OPV) consists of three live attenuated strains that correspond to the three serotypes of poliovirus (Sabin 1, 2, and 3, for Polio 1, 2, and 3, respectively) [1]. PVs were the main causes of paralytic disease (poliomyelitis) before PV vaccines were available. However, poliomyelitis has not been absolutely eradicated, because the poliomyelitis eradication program is based on a large-scale vaccination campaign with OPV and the vaccine-derived polioviruses (VDPVs) or vaccine-related polioviruses (VRPVs) have caused sporadic or epidemics of poliomyelitis in many countries [2–6].

During the replication of Sabin strains in gut cells, revertants with increased virulence and recombination (gene rearrangements) can arise, leading to the emergence of pathogenic VDPVs or VRPVs, which have been found in patients with acute flaccid paralysis (AFP) [2, 4, 7]. Most recombinants were found among Sabin 2 and Sabin 3 [8–12], and a small proportion of recombinants were found among Sabin 1 with Sabin 2 or wild vaccine [13]. Crossover sites have been found in genomic regions coding for non-structural and structural proteins [8–13].

Here, we report four VRPV3 strains, including two type 3/type 2 intertypic recombinants, isolated from aseptic encephalitis cases in 2010, China. This finding prompted us to further explore the complete genome characterization and temperature-sensitive phenotype of the four VRPV3 isolates.

## Results

### Primary characterization of the virus isolates

The entire VP1 coding sequences of RF108/YN/CHN/2010 (RF108), RF134/YN/CHN/2010 (RF134), RF146/YN/CHN/2010 (RF146) and RF151/YN/CHN/2010 (RF151) were found to have high homology with PV3/Sabin (99.8 %). PV3 isolates that are <1.0 % divergent in the VP1 sequence from PV3/Sabin are classified as VRPV [14], the four isolates belong to VRPV3. Neutralization assay tests on all four isolates with anti-PV1, 2 and 3 antisera, respectively, revealed that all four isolates were completely neutralized with polyclonal anti-PV3 antisera but not by anti-PV1 or anti-PV2 antisera, this further determined a PV3/Sabin serotype.

### Analysis of complete genome sequences

Excluding a small fragment (29 nucleotides) at the 5′-end of the genomes, the complete genome sequences of the four VRPV3 isolates were determined. The genomes were 7402 nucleotides in length and consisted of a 5′-UTR of 713 nucleotides followed by a long ORF that encoded a polyprotein of 2206 amino acids and a 3′-UTR of 71 nucleotides. The variation between these four strains at the nucleotide and amino acid level were 0.2–4.9 % and 0.2–1.2 %, respectively.

The 3A to 3′-UTR regions of RF108 and 3′-UTR regions of RF134, RF146 and RF151 had most homology with the corresponding regions of Sabin 2, while other regions of their genomes had high similarity to the corresponding regions of Sabin 3 (Table 1). Importantly, the two major determinants for the attenuated phenotype of Sabin 3 were not lost in the four isolates: T472 in the 5′-UTR and U2034 (also identified as Phe91) in the VP3 region [15]. Another Sabin 3-specific mutation at position 2493 (T → C), which predicts an Ile → Thr (at residue 6 of VP1) change that was found to have attenuating neurovirulence in monkeys [16], had reverted back to a T in all four isolates. Except for in the Sabin 3 and PV3 NIE1018453 strain, the position T2493 of other PV3 isolates was identical to the wild poliovirus type 3 (WPV3) strain P3/Leon/37(type 3) (Table 2).

**Table 1:** Pairwise nucleotide (amino acid) % identity between Sabin 1, Sabin 2 and Sabin 3 and 4 PV3 isolates in all sequenced genomic regions

Genomic region	RF108			RF134			RF146			RF151		
	Sabin 1	Sabin 2	Sabin 3	Sabin 1	Sabin 2	Sabin 3	Sabin 1	Sabin 2	Sabin 3	Sabin 1	Sabin 2	Sabin 3
5′UTR	80.3	80.4	99.9	80.3	80.4	99.9	80.3	80.4	99.9	80.0	80.2	99.6
VP4	77.3 (92.8)	75.2 (90.6)	100 (100)	77.3 (92.8)	75.2 (92.6)	100 (100)	77.3 (92.8)	75.2 (92.6)	100 (100)	77.3 (92.8)	75.2 (92.6)	100 (100)
VP2	70.3 (82.4)	70.2 (81.2)	99.9 (100)	70.3 (82.4)	70.2 (81.2)	99.9 (100)	70.3 (82.4)	70.2 (81.2)	99.9 (100)	70.5 (82.7)	70.2 (81.2)	99.9 (100)
VP3	72.4 (84.5)	73.4 (86.1)	99.7 (99.2)	72.1 (84.0)	73.4 (86.6)	100 (100)	72.1 (84.0)	73.4 (86.6)	100 (100)	72.1 (84.0)	73.4 (86.6)	100 (100)
VP1	65.1 (74.9)	68.8 (78.5)	99.8 (99.3)	65.1 (74.9)	68.5 (78.5)	99.8 (99.3)	65.3 (75.6)	68.8 (78.5)	99.8 (99.3)	65.1 (74.9)	68.8 (78.5)	99.8 (99.3)
2A	80.8 (94.0)	78.7 (93.3)	99.8 (100)	80.8 (94.0)	78.7 (93.3)	99.8 (100)	80.8 (94.0)	78.7 (93.3)	99.8 (100)	80.8 (94.0)	78.7 (93.3)	99.8 (100)
2B	76.3 (91.8)	81.8 (94.8)	100 (100)	76.3 (91.8)	81.8 (94.8)	100 (100)	76.3 (91.8)	81.8 (94.8)	100 (100)	76.3 (91.8)	81.8 (93.3)	100 (100)
2C	82.1 (97.6)	87.5 (97.6)	97.5 (98.8)	82.8 (97.0)	85.1 (96.4)	99.9 (100)	82.7 (97.0)	85.2 (96.4)	99.8 (100)	82.7 (97.0)	85.1 (96.4)	99.9 (100)
3A	82.8 (98.9)	100 (100)	82.4 (97.7)	85.4 (98.9)	82.4 (97.7)	100 (100)	85.4 (98.9)	82.4 (97.7)	100 (100)	85.4 (98.9)	82.4 (97.7)	100 (100)
3B	83.3 (95.5)	100 (100)	86.4 (95.5)	86.4 (90.9)	84.8 (95.5)	98.5 (100)	86.4 (90.9)	84.4 (95.5)	98.5 (100)	86.4 (90.9)	84.8 (95.5)	98.5 (100)
3C	86.7 (98.4)	99.6 (100)	86.5 (97.3)	89.6 (98.9)	86.5 (97.3)	100 (100)	89.6 (99.5)	86.5 (97.8)	99.6 (99.5)	89.6 (99.5)	86.5 (97.3)	100 (100)
3D	87.1 (97.8)	99.9 (100)	85.8 (97.6)	86.0 (97.8)	92.0 (97.8)	93.5 (99.8)	85.3 (97.0)	85.6 (97.0)	99.6 (99.3)	85.4 (97.4)	92.0 (97.8)	93.5 (99.8)
3′UTR	91.5	95.7	93.0	95.8	100	97.2	91.5	95.7	93.0	91.5	100	97.2
Genome	78.5 (90.7)	84.0 (92.0)	95.1 (98.8)	78.8 (90.5)	80.5 (91.0)	98.7 (99.9)	78.6 (90.5)	79.3 (90.9)	99.7 (99.7)	78.6 (90.50	79.1 (91.0)	99.7 (99.7)

**Table 2:** Nucleotide and amino acid substitutions of attenuation positions in oral PV vaccine strains Sabin 3 and four PV3 isolates

Strain	5′UTR	VP3		VP1	
	Nucleotide (472)	Nucleotide (2034)	Amino acid (91)	Nucleotide (2493)	Amino acid (6)
Sabin 3	T	T	Phe	C	Thr
RF108	T	T	Phe	T	ILe
RF134	T	T	Phe	T	ILe
RF146	T	T	Phe	T	ILe
RF151	T	T	Phe	T	ILe
WPV3	C	C	Ser	T	ILe

The nucleotide mutation position refers to the complete genome, whereas the corresponding amino acid change is numbered according to the primary amino acid sequence of a given protein after cleavage of the polyprotein precursor

The amino acid sequences within or near the predicted neutralizing antigenic (NAg) sites were identified in an alignment of the four isolates, Sabin 3, Sabin 2 and 16 VRPV3 strains and P3/Leon/37(type 3). Except for the RF151 strain, there were no amino acid substitutions at NAg sites in the other three isolates (Additional file 1: Figure S1). Sabin-specific antigenic epitopes were present in the three isolates, suggesting that RF108, RF134, and RF146 isolates were antigenically indistinguishable from Sabin 3.

### Phylogenetic analysis

**Fig. 1:**
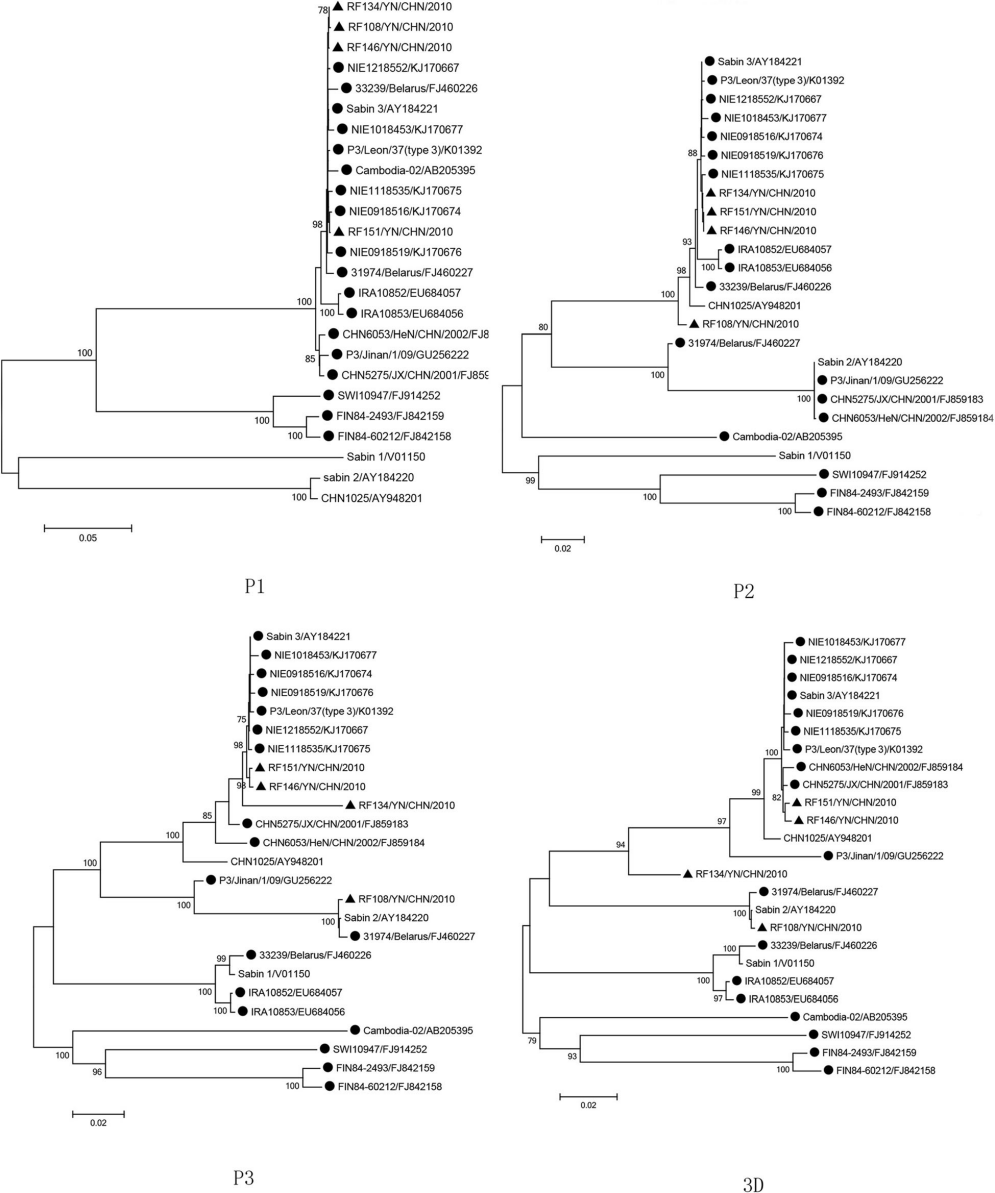
Phylogenetic trees based on the P1, P2, P3 and 3D genomic regions generated by the neighbor-joining algorithm implemented in MEGA (version 6.06) using the Kimura two-parameter substitution model and 1,000 bootstrap pseudo-replicates. ▲strains isolated in this investigation; ● other PV3 strains

Phylogenetic trees were constructed based on the P1, P2, P3 and 3D genomic regions, respectively, with 22 PV3 (including RF134, RF151, RF146 and RF108) strains and one Sabin 2 strain (Fig. 1). In the regions of P1 and P2, the four isolates in the study clustered together with most PV3 isolates (including Sabin 3), but in P2, one PV3/PV2 intertypic 3D-recombinant PV (31974/Belarus) [17] and three PV3/PV2 intertypic VP1 capsid-recombinant PV3 strains (P3/Jinan/1/09, CHN6053/HeN/CHN/2002 and CHN5275/JX/CHN/2001) [12] clustered with Sabin 2. In the P1, one intertypic PV2/PV3 penta-recombinant PV2 (CHN1025, five successive rounds of recombination occurred in the VP1 capsid coding region and in the 2C, 3C (twice) and 3D coding regions [11] clustered with Sabin 2. In the P3 and 3D, RF108 clustered with Sabin 2 and 31974/Belarus, while RF134, RF146, and RF151 were grouped into a cluster of the indigenous Sabin 3 and most of the PV3 isolates. However, the nucleotide identity of RF134 was not significantly high (93.5 %), and RF134 could belong to another subclade. Besides, phylogenetic analysis of 3D genomic regions on all HEV-C reference strains could also indicate recombination with other HEV-C serotypes as two PV3/PV1 intertypic 2C-recombinant PVs (IRA10852 and RA10853) [2], one PV3/PV1 intertypic 3D-recombinant PV(3239/Belarus) [17], and four PV3/HEV-C intertypic 3D-recombinant PVs (FIN84-2493, FIN84-60212, Cambodia-02 and SWI10947) [2, 18] (Additional file 2: Figure S2). The aforementioned results clearly show that the phylogenetic analysis of different genomic regions could indicate recombination positions between enteroviruses.

### Recombination analysis

**Fig. 2:**
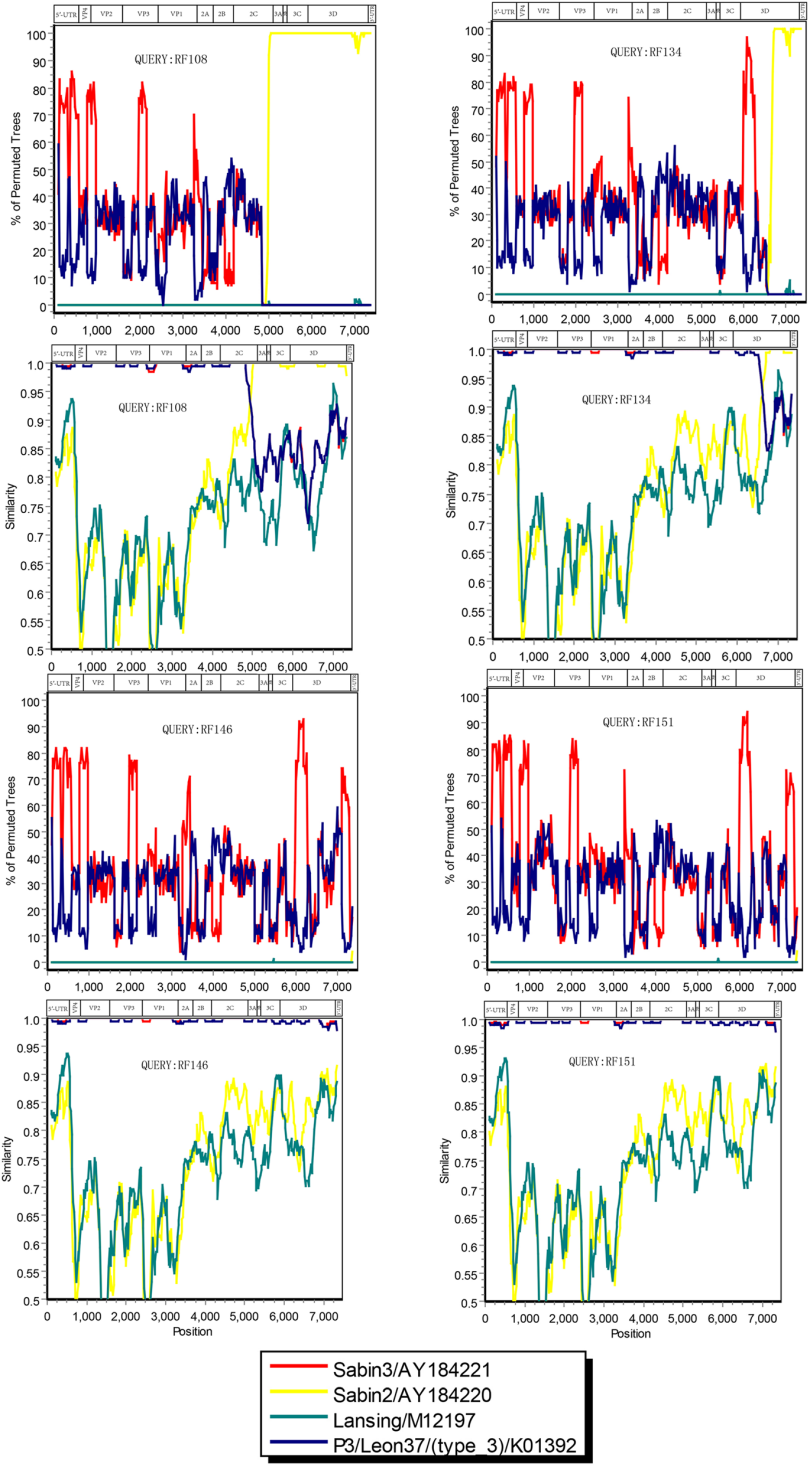
Similarity plot and bootscanning analyses of the complete genome of 4 PV3 isolates using a sliding window of 200 nt moving in 20-nt steps. The names of the viruses used as the reference strains were indicated in the square and the names of viruses of the query sequence were indicated in the middle

The above recombination hypothesis was further examined by Simplot and bootscanning analyses in Fig. 2. Two of the four VRPVs (strains RF108, RF134, RF146, and RF151), RF108 and RF134 strains, were found to have recombination in one crossover site: 5′-UTR to 5′-terminal of the 2C coding region and 5′-UTR to 5′-terminal of the 3D coding region of the genome were the Sabin 3 sequence and the 3′ part was the Sabin 2 sequence (PV3/PV2), respectively. The crossover site was located in the 2C and 3D coding region (nt 4941 and 6621), respectively. In other words, the RF108 and RF134 isolated from different patients indicated diverse mosaic recombinant genomic structures composed of sequences derived from Sabin 3 and from Sabin 2 (Fig. 2). The findings described above suggested that RF108 and RF134 strains from Yunnan, China were PV3/PV2 recombinant strains.

### Temperature sensitivity

All four isolates were compared to Sabin 3 with regards to replication capacity at 36 °C and 40 °C. The difference in titers between the two temperatures was reduced significantly more than 2 logarithms for all isolates as with Sabin 3 (Additional file 3: Table S1). This indicates that the four isolates were temperature-sensitive.

## Discussion

Through the effective large-scale use of OPV, WPV3 has not been detected around the world since 2012 [19]. There were many reports of AFP cases caused by VDPV3 [10–12, 20–22], but AFP caused by VRPV3 has rarely been reported. To date, only one report has described AFP caused by VRPV3, which was a recombinant [2]. In this study, four Sabin 3-related PVs (RF108, RF134, RF146, and RF151) were isolated from aseptic encephalitis cases in Yunnan, China in 2010, which were classified as VRPVs by divergence (<0.6 %) in VP1 complete sequences and genetically unrelated to each other [22]. Therefore, this result showed that the four isolates did not result from a circulating strain [2].

In addition, all four isolates demonstrated most characteristics of Sabin 3, such as the same NAg sites (except RF146), two attenuated nucleotide positions (T472 and T2034) [15], and a temperature-sensitive phenotype. The T2034 (at Phe-91of VP3) was also a major determinant of temperature-sensitivity [23]. Besides this residue, another 11 amino acid substitutions were suggested to be related to temperature sensitivity [23], and mutations of those amino acids are also found in the four isolates (data not shown). So, the molecular basis also shows that the four isolates possess the same temperature-sensitive phenotype as Sabin 3. Furthermore, a Thr-6 → Ile substitution of VP1 (nucleotide 2493) which has been frequently found in VDPV3 and WPV3 strains appeared in all four isolates.

Recombination of PV3 also occurs in genome regions as with HEV-Cs, including different serotypes of PVs, and the crossover sites of recombinants may be located in the P1, P2 or P3 coding region [2, 8, 24–28]. The 3′-end of the 2C to 3′-UTR genome region of RF108 and 5′-end of 3D to 3′-UTR genome region of RF134 had most homology with Sabin 2, respectively. This indicated the crossover site of the two PV3 recombinants may be located in the P2 and P3 genome regions. The 3D phylogenetic tree showed that RF108 was most relevant to Sabin 2, suggesting genetic rearrangement had occurred between these two strains. Interestingly, although RF134 was not sufficiently relevant to Sabin 2, our similarity plot and bootscanning analyses of the genomic sequences of the RF134 strain and Sabin 2 showed recombination sites in the nonstructural coding regions (Fig. 2), recombination events were observed between the RF108 or RF134 strain with Sabin 2, with support values of more than 90 %. Obviously, recombination of the RF108 or RF134 isolates had occurred with Sabin 2. It was clear that the 3D phylogenetic analyses hinted at recombination between enteroviruses as reported previously [29]. In previous reports of HEV-C 3D phylogenetic trees, recombination strains with other HEV-C strains could be determined: Cambodia-02, SWI10947, FIN84-60212 and FIN84-2493 [2, 18]. However, the 3D phylogenetic tree does not cover recombination that has occurred in structural coding regions such as VP1 (as in P3/Jinan/1/09, CHN5275/JX/CHN/2001, and CHN6053/HeN/CHN/2002). So, the phylogenetic trees based on the different coding regions could not only ascertain recombinants, they could also find crossover genomic regions.

According to VRPV3 that differs from the OPV3 strain by >1 % of nucleotide positions are estimated to have replicated in one or more individuals for at least one year after administration of an OPV dose [22], the four strains were isolated within six months after the administration of OPV. So, the four strains should have kept the most original genetic characteristics of Sabin 3 [2].

## Conclusion

In this study, we have identified four VRPV3 strains, including two type 3/type 2 intertypic recombinants, associated with aseptic encephalitis. It is possible that VRPVs and VDPVs still exist for a long period until OPV is completely replaced with inactivated poliovirus vaccine. VRPVs or VDPVs are not only often associated with recombination with different Sabin serotypes but also non-Sabin enteroviruses [2, 30]. The recombination of Sabin vaccine strains with other serotype or HEV-C would be a critical factor for the potential of emerging viruses and related disease outbreaks. Thus, it is essential to persistent surveillance of EVs (including PV).

## Methods

### Stool specimens and virus isolation

The PV3 RF108, RF134, RF146 and RF151 strains were isolated from four fecal specimens of a five-year-old boy, four-year-old boy, five-year-and-nine-month-old girl and four-year-old girl, respectively (Additional file 4: Table S2), in 2010 during an enterovirus surveillance in Yunnan, China.

All of the children with aseptic meningitis received at least three doses of OPV after birth. They did not show signs of immunodeficiency at the time of presentation. RD, KMB17, and A549 cell lines were used to isolate viruses from the stool specimens according to standard procedures [31]. All positive isolates were stored at −80 °C.

### Viral RNA extraction, RT-PCR, sequencing and typing

The viral RNAs were extracted from cell culture supernatants with a QIAamp Viral RNA Mini Kit (QIAGEN, Valencia, CA, USA) according to the manufacturer’s instructions. RT-PCR was carried out using a PrimeScript™ One Step RT-PCR Kit Ver.2 (TaKaRa, Dalian, China) according to the manufacturer’s protocol. The primer pairs 222 and 224 were used to amplify the partial VP1 gene [32]. The partial VP1 sequences were compared with sequences from GenBank using BLAST (http://www.ncbi.nlm.nih.gov/BLAST/). In addition, all isolates were identified by a microneutralization test with poliovirus type-specific rabbit polyclonal antisera (Chinese Academy of Medical Sciences and Peking Union Medical College Institute of Medical Biology, Kunming, China). The design of primers to amplify the complete genomes were based on the published sequence of Sabin 3 and primers were designed for “primer-walking” strategy as described previously [31] (Additional file 5: Table S3). The PCR positive products were sequenced using an ABI 3730XL automatic sequencer (Applied Biosystems, Foster City, CA, USA) at BGI Sequencing Company (Beijing, China). Nucleotide sequences used in this study have been deposited in GenBank under accession numbers KT946714–KT946717.

### Sequence analysis

Phylogenetic trees were conducted using Molecular Evolutionary Genetic Analysis (MEGA) version 6.06 software as described previously [32]. The plot of nucleotide similarities was created using Simplot software version 3.5.1, with a sliding window of 200 nucleotides moving in steps of 50 nucleotides [32]. Pairwise alignments of the sequences were performed using Geneious Basic 5.6.5 software [32].

### Temperature sensitivity

The temperature sensitivity of the four PV3 isolates was assayed on a monolayer of Hep2 (human laryngeal tumor cells). Two 24-well plates were inoculated with 50 μl of undiluted virus stocks (Sabin 3 and four isolates). After absorption at 36 °C or 40 °C for 1 h, the unabsorbed virus was removed, maintenance medium was added to each well and the plates were continually incubated at 36 °C or 40 °C. After 8 h and 24 h of post-infection, the cultural supernatants were harvested, respectively and the cell culture infectious dose 50 % was calculated by the end-point dilution method in 96-well plates at 36 °C. The isolate showing more than two logarithms reduction of the titers at 36 °C or 40 °C was considered to be temperature sensitive [9].

## Additional files

Additional file 1: Figure S1. Alignment of amino acid residues of neutralizing antigenic (NAg) sites 1 (VP1: 88–106), 2 (VP2: 163–169; VP2: 268–270; VP1:220–225), 3a (VP3: 54–61; VP3: 70–74; VP1: 286–291), and 3b (VP2: 71–73; VP3: 75–79) for Sabin 3. (DOC 408 kb) Additional file 2: Figure S2. Phylogenetic trees based on 3D genomic regions of HEV-C generated by the neighbor-joining algorithm implemented in MEGA (version 6.06) using the Kimura two-parameter substitution model and 1,000 bootstrap pseudo-replicates. ▲strains isolated in this investigation; ● other PV3 strains. (DOC 682 kb) Additional file 3: Table S1. Temperature sensitivity of 4 poliovirus type 3 isolates. (DOC 31 kb) Additional file 4: Table S2. Primary characterization of 4 PVs isolated from viral encephalitis case patients. (DOC 29 kb) Additional file 5: Table S3. Primers used for RT-PCR and sequencing of the PV 3 isolates genome. (DOC 39 kb)

## Abbreviations

HEV-C: Human enterovirus C species
OPV: Oral poliovirus vaccine
ORF: Open reading frame
PVs: Polioviruses
VDPVs: Vaccine-derived polioviruses
VRPVs: Vaccine-related polioviruses
