# Supplementary material for: Characterization of four vaccine-related polioviruses including two intertypic type 3/type 2 recombinants associated with aseptic encephalitis
Source: Virol J. 2016 Sep 27;13:162. doi: 10.1186/s12985-016-0615-2 (PMC5039789; doi:10.1186/s12985-016-0615-2)
Supplement: Additional file 1: Figure S1. — Alignment of amino acid residues of neutralizing antigenic (NAg) sites 1 (VP1: 88–106), 2 (VP2: 163–169; VP2: 268–270; VP1:220–225), 3a (VP3: 54–61; VP3: 70–74; VP1: 286–291), and 3b (VP2: 71–73; VP3: 75–79) for Sabin 3. (DOC 408 kb) [file 12985_2016_615_MOESM1_ESM.doc]

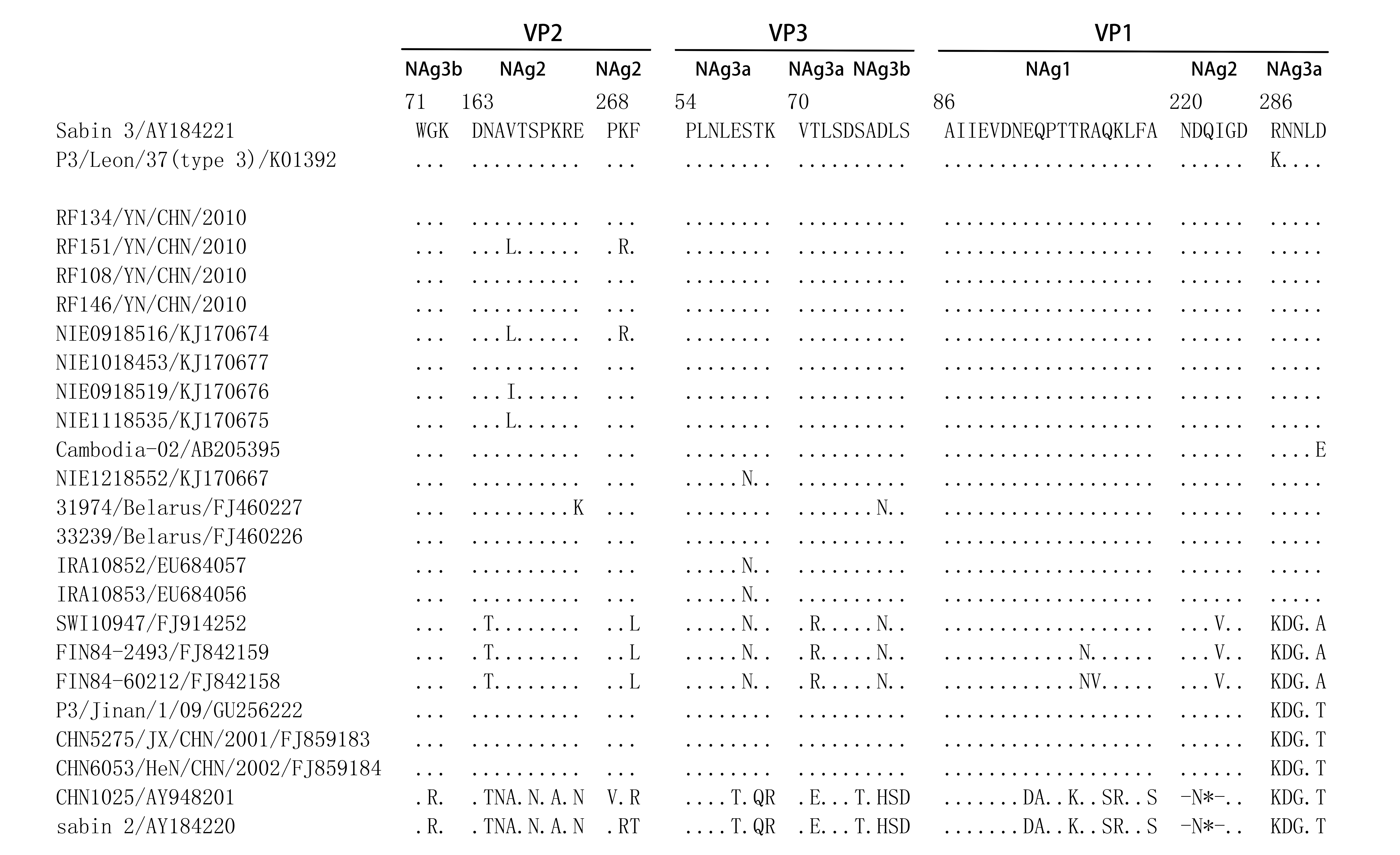


Additional file 1: **Figure S1**. Alignment of amino acid residues of neutralizing antigenic (NAg) sites 1 (VP1: 88–106), 2 (VP2: 163–169; VP2: 268–270; VP1:220–225), 3a (VP3: 54–61; VP3: 70–74; VP1: 286–291), and 3b (VP2: 71–73; VP3: 75–79) for Sabin 3.
