# Supplementary material for: Characterization of four vaccine-related polioviruses including two intertypic type 3/type 2 recombinants associated with aseptic encephalitis
Source: Virol J. 2016 Sep 27;13:162. doi: 10.1186/s12985-016-0615-2 (PMC5039789; doi:10.1186/s12985-016-0615-2)
Supplement: Additional file 2: Figure S2. — Phylogenetic trees based on 3D genomic regions of HEV-C generated by the neighbor-joining algorithm implemented in MEGA (version 6.06) using the Kimura two-parameter substitution model and 1,000 bootstrap pseudo-replicates. ▲strains isolated in this investigation; ● other PV3 strains. (DOC 682 kb) [file 12985_2016_615_MOESM2_ESM.doc]

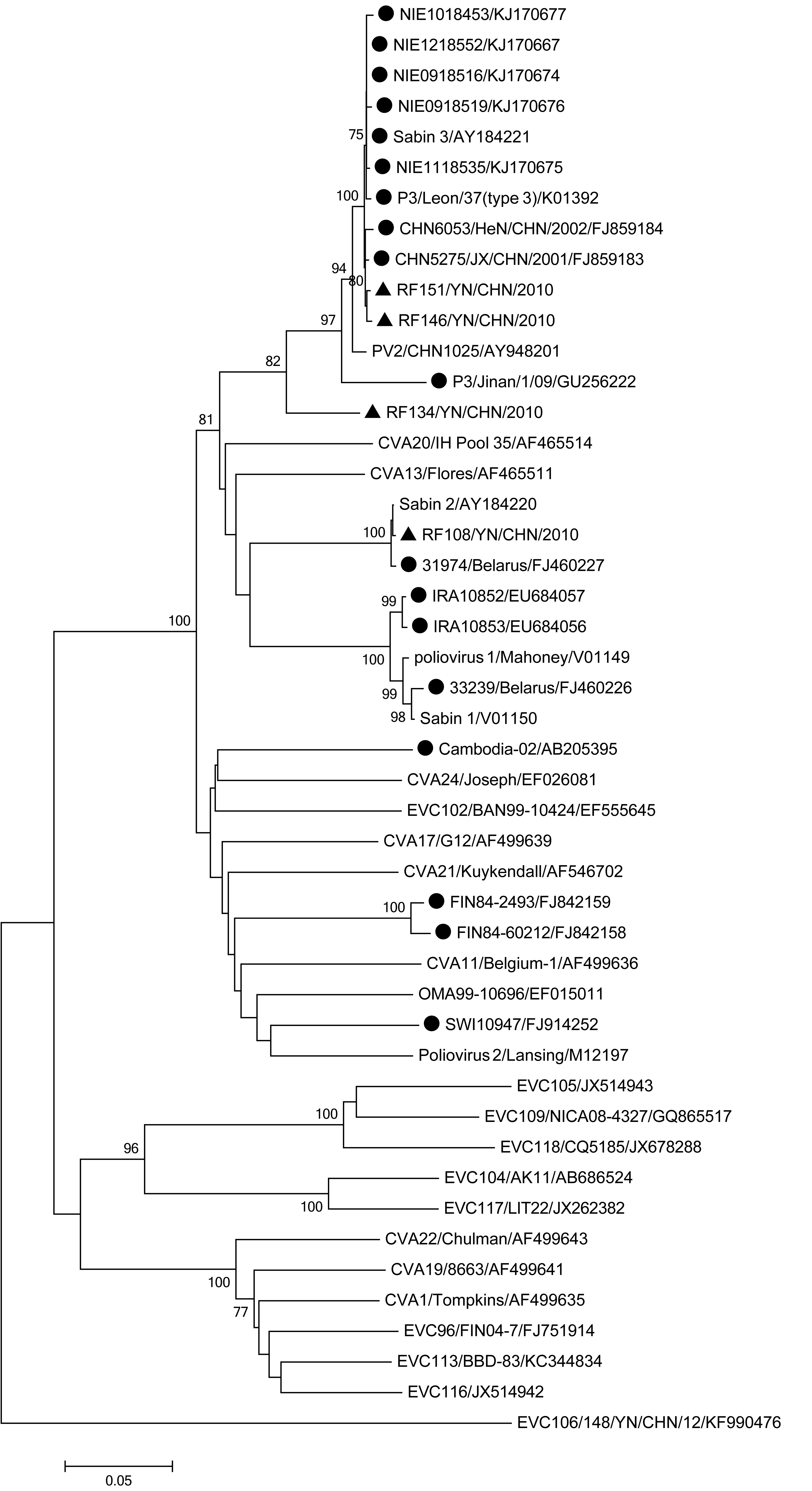


Additional file 2: **Figure S2**. Phylogenetic trees based on 3D genomic regions of HEV-C generated by the neighbor-joining algorithm implemented in MEGA (version 6.06) using the Kimura two-parameter substitution model and 1,000 bootstrap pseudo-replicates. ▲strains isolated in this investigation; ● other PV3 strains.
