# Supplementary material for: Characterization of four vaccine-related polioviruses including two intertypic type 3/type 2 recombinants associated with aseptic encephalitis
Source: Virol J. 2016 Sep 27;13:162. doi: 10.1186/s12985-016-0615-2 (PMC5039789; doi:10.1186/s12985-016-0615-2)
Supplement: Additional file 3: Table S1. — Temperature sensitivity of 4 poliovirus type 3 isolates. (DOC 31 kb) [file 12985_2016_615_MOESM3_ESM.doc]

Additional file 3: **Table S1**. Temperature sensitivity of 4 poliovirus type 3 isolates

| Growth temperature and virus titer | | | | | | | | |
| --- | --- | --- | --- | --- | --- | --- | --- | --- |
| Virus strain | 36℃  (X±SD) | | 40℃  (X±SD) | | Log titer reduction 36℃/40℃  (d±SD) | | P value | |
|  | 8h p.i | 24h p.i | 8h p.i | 24h p.i | 8h p.i | 24h p.i | t | P |
| Sabin 3 | 5.538±0.066 | 6.917±0.072 | 2.750±0.125 | 3.583±0.144 | 2.788±0.185 | 3.333±0.072 | 8.400 | 0.0000232875 |
| RF108 | 6.208±0.072 | 6.750±0.125 | 3.417±0.072 | 3.708±0.072 | 2.792±0.072 | 3.042±0.191 | 20.124 | 0.0000000517 |
| RF134 | 5.792±0.072 | 6.208±0.072 | 3.708±0.072 | 4.083±0.072 | 2.083±0.144 | 2.125±0.000 | 16.091 | 0.0000000178 |
| RF146 | 6.000±0.125 | 6.125±0.125 | 3.538±0.066 | 3.208±0.072 | 2.462±0.075 | 2.917±0.144 | 28.424 | 0.0000000001 |
| Rf151 | 6.000±0.650 | 6.000±0.125 | 3.300±0.130 | 3.125±0.000 | 2.700±0.595 | 2.875±0.125 | 14.852 | 0.0000027861 |

Note: Values are log10 TCID_50_ /ml virus produced in Hep2 cells at 36 °C and 40°C. Statistical analysis was performed using SPSS (Statistical Package for the Social Sciences) 12.0 software (Chicago, IL, USA). Two-tailed *P-*values <0.05 were considered significant.
