# Supplementary material for: Characterization of four vaccine-related polioviruses including two intertypic type 3/type 2 recombinants associated with aseptic encephalitis
Source: Virol J. 2016 Sep 27;13:162. doi: 10.1186/s12985-016-0615-2 (PMC5039789; doi:10.1186/s12985-016-0615-2)
Supplement: Additional file 4: Table S2. — Primary characterization of 4 PVs isolated from viral encephalitis case patients. (DOC 29 kb) [file 12985_2016_615_MOESM4_ESM.doc]

Additional file 4: **Table S3**. Primary characterization of 4 PVs isolated from viral encephalitis case patients

| Virus  isolates | Age(yr)/ Sex | OPV history | Dates of  Last OPV Onset Sampling | | |
| --- | --- | --- | --- | --- | --- |
| RF108 | 5.5/M | 4 | Unknown | 30/Mar/2010 | 30/Mar/2010 |
| RF134 | 4/M | 4 | 5/Jan/2010 | 17/Apr/2010 | 17/Apr/2010 |
| RF146 | 5.9/M | 4 | Unknown | 28/Apr/2010 | 28/Apr/2010 |
| RF151 | 4/F | 4 | 5/Dec/2009 | 29/Apr/2010 | 1/May/2010 |
