# Supplementary material for: Characterization of four vaccine-related polioviruses including two intertypic type 3/type 2 recombinants associated with aseptic encephalitis
Source: Virol J. 2016 Sep 27;13:162. doi: 10.1186/s12985-016-0615-2 (PMC5039789; doi:10.1186/s12985-016-0615-2)
Supplement: Additional file 5: Table S3. — Primers used for RT-PCR and sequencing of the PV 3 isolates genome. (DOC 39 kb) [file 12985_2016_615_MOESM5_ESM.doc]

Additional file 5: **Table S2**. Primers used for RT-PCR and sequencing of the PV 3 isolates genome

Note:F or f (forward) and R (reverse) mean the orientation of primer;

| Name | Sequence(5’→ 3’) | Position |
| --- | --- | --- |
| Pol31F | CAGAGGCCCACGTGGCGGCT | 30-49 |
| Pol31R | CATTACACTGCACGTGCACA | 1260-1241 |
| Pol32F | CTGAACCAGATGTGGCTACA | 1081-1100 |
| Pol32R | GAGTGGTGTCTCGCAGCAAT | 2450-2431 |
| 222 | CICCIGGIGGIAYRWACAT | 2969-2951 |
| 224 | GCIATGYTIGGIACICAYRT | 1977-1996 |
| Pol34F | AGAATCATTCTTCGCACGCG | 2672-2691 |
| Pol34R | CCTCCATGTATTGGAAGGTG | 3600-3581 |
| Pol35F | GATCTGCAACTACCATCT | 3392-3409 |
| Pol35R | TTAACTGAATTGCCTTGCC | 5000-4981 |
| Pol36F | TTTGACGTGGACATACAAG | 4851-4870 |
| Pol36R | TTGTTCCCTACATACTTA | 6145-6128 |
| Pol37F (RF108) | CCCTTGAAGATCAGGCAG | 5618-5636 |
| Pol37R | GGAGACTAGCGTCAACCT | 6975-6958 |
| Pol38F | GTGGCCATGAGAATGGCATT | 6501-6520 |
| Pol38R | CCCCGAATTAAAGAAAAATT | 7439-7420 |
| Pol36F(RF134) | CATCACACCACCTACAGTAG | 4763-4782 |
| Pol26R(RF108) | CATCATAACCTGTGTAGT | 6700-6683 |
